# Quality changes of workplace health promotion in Austrian companies over time

**DOI:** 10.1093/eurpub/ckac129.587

**Published:** 2022-10-25

**Authors:** G Lang, C Heigl, P Jiménez

**Affiliations:** 1 Austrian Health Promotion Fund, Austrian National Public Health Institute, Vienna, Austria; 2 Austrian Health Insurance Fund, Austrian Network for Workplace Health Promotion, Linz, Austria; 3 University of Graz, Institute of Psychology, Graz, Austria

## Abstract

**Background:**

Workplace health promotion (WHP) is effective when it is implemented in a high-quality and sustainable manner. Companies in the Austrian quality management system can apply for a WHP quality certificate every three years. More and more companies are integrating WHP into their regular operations. This work investigates the WHP quality system and how the companies develop over time.

**Methods:**

WHP quality is measured using 15 holistic quality criteria, which are assessed by an external, independent institute. For the period 2014-2021, evaluations from n = 570 companies with two and from n = 278 companies over three measurement points in time are available (initial and renewal awards). The (potential) change of the WHP quality is examined with a longitudinal design by the means of confirmatory factor analyses and autoregressive models with latent variables.

**Results:**

The measurement of WHP quality shows acceptable to good measurement accuracy (internal consistency-reliability: α = 0.85-0.89), validity (convergent and discriminant validity) and metric measurement invariance over time. At structural level, relative stability of WHP quality can be demonstrated for the measurement points (β = 0.40-0.52, p < 0.001). Both the quality of WHP and the temporal stability differentiate significantly according to the firm size and the experience of the company with WHP structures and processes.

**Conclusions:**

The use of the criteria allows a reliable, valid and objective quality assessment of WHP quality due to the concept of the assessment process. WHP quality is a relatively stable characteristic of the company, which, however, varies according to temporal, structural and process-related aspects. Company size moderates to a lesser extent of quality changes over time. This is in line with expectations, but also indicates that small companies should be clearly motivated and supported for their WHP measures. The insights help to further develop quality assurance in WHP and public health.

**Key messages:**

• The criteria used allow a reliable, valid and objective assessment of WHP quality.

• Because firm size moderates the change in WHP quality, small firms should be continuously encouraged and supported.

